# China’s community-based strategy of universal preconception care in rural areas at a population level using a novel risk classification system for stratifying couples´ preconception health status

**DOI:** 10.1186/s12913-016-1930-4

**Published:** 2016-12-28

**Authors:** Qiongjie Zhou, Shikun Zhang, Qiaomei Wang, Haiping Shen, Weidong Tian, Jingqi Chen, Ganesh Acharya, Xiaotian Li

**Affiliations:** 1 Obstetrics and Gynecology Hospital of Fudan University, 419 Fangxie Road, Shanghai, 200011 China; 2 The Shanghai Key Laboratory of Female Reproductive Endocrine-Related Diseases, Shanghai, China; 3 Women’s Health and Perinatology Research Group, Department of Clinical Medicine, UiT - The Arctic University of Norway, Tromsø, Norway; 4 The National Health and Family Planning Commission, Beijing, China; 5 School of Life Sciences, Institute of Biostatistics, Fudan University, Shanghai, China; 6 Department of Clinical Science, Intervention and Technology (CLINTEC), Karolinska Institute, Stockholm, Sweden; 7 Institute of Biomedical Sciences, Fudan University, Shanghai, China

**Keywords:** Preconception care, Preconception health, Risk stratification, Reproductive health, Population-based study, Rural China, Universal preconception care, Community-based care

## Abstract

**Background:**

Preconception care (PCC) is recommended for optimizing a woman’s health prior to pregnancy to minimize the risk of adverse pregnancy and birth outcomes. We aimed to evaluate the impact of strategy and a novel risk classification model of China´s “National Preconception Health Care Project” (NPHCP) in identifying risk factors and stratifying couples’ preconception health status.

**Methods:**

We performed a secondary analysis of data collected by NPHCP during April 2010 to December 2012 in 220 selected counties in China. All couples enrolled in the project accepted free preconception health examination, risk evaluation, health education and medical advice. Risk factors were categorized into five preconception risk classes based on their amenability to prevention and treatment: **A**-avoidable risk factors, **B**- benefiting from targeted medical intervention, **C**-controllable but requiring close monitoring and treatment during pregnancy, **D**-diagnosable prenatally but not modifiable preconceptionally, **X**-pregnancy not advisable. Information on each couple´s socio-demographic and health status was recorded and further analyzed.

**Results:**

Among the 2,142,849 couples who were enrolled to this study, the majority (92.36%) were from rural areas with low education levels (89.2% women and 88.3% men had education below university level). A total of 1463266 (68.29%) couples had one or more preconception risk factors mainly of category A, B and C, among which 46.25% were women and 51.92% were men. Category A risk factors were more common among men compared with women (38.13% versus 11.24%; *P* = 0.000).

**Conclusions:**

This project provided new insights into preconception health of Chinese couples of reproductive age. More than half of the male partners planning to father a child, were exposed to risk factors during the preconception period, suggesting that an integrated approach to PCC including both women and men is justified. Stratification based on the new risk classification model demonstrated that a majority of the risk factors are avoidable, or preventable by medical intervention. Therefore, universal free PCC can be expected to improve pregnancy outcomes in rural China.

## Background

Preconception care (PCC) is defined as interventions that aim to identify and, when possible, modify the biomedical, behavioral, and social risks to optimize woman’s health before pregnancy with the aim of improving pregnancy outcomes [1]; In 2014, Centers for Disease Control and Prevention (CDC) and the Office of Population Affairs published clinical recommendations, “Providing Quality Family Planning Services” (QFP), and recognized PCC as a critical component of health care for women of reproductive age [2].

The purpose of PCC is to optimize a woman’s health prior to pregnancy and promote healthy behavior during pregnancy to reduce the incidence of adverse birth outcomes [3]. It is reported that an estimated 300,000 women die globally as a result of pregnancy-related conditions [4]. The prevalence of birth defects in China is around 5.6%, and there are nearly 900 000 new cases annually according to the official Report on Prevention of Birth Defects in China published in 2012 [5]. Health services provided to the couples of reproductive age, such as family planning, folic acid supplementation [6], genetic counseling, chronic disease management, immunizations, treatment of sexually transmitted infections, and interventions promoting healthier lifestyle, including those directed against alcohol, tobacco, and substance abuse [7] seem to have a positive effect. There is growing evidence that effective treatment of maternal diabetes and hypertension during the preconception period reduces adverse maternal and neonatal outcomes [8–10]. Avoiding unintended pregnancy through PCC could avert 44% maternal mortality [11]. Moreover, the effect of PCC on women with a history of previous adverse infant outcome, such as preterm birth, low birth weight, stillbirth or major birth defect, appears to be meaningful [12].

Even though the benefits of PCC have been well established [13, 14], integrating PCC into regular family planning services still remains a challenge for some providers [15]. Poor organization of health services’ delivery systems, lack of comprehensive PCC programs, limited awareness among future parents about the availability and benefits of PCC and that of physicians about the necessity and effectiveness of PCC are apparent barriers affecting delivery and uptake of PCC [16, 17].

PCC in China has been insufficient and inadequate, especially in rural areas, despite the fact that facility-based strategy on reducing neonatal mortality had a significant impact on the Millennium Development Goal 4, and with a rapid economic development there have been improvements in population health in recent decades [18]. Therefore, the National Health and Family Planning Commission of the People’s Republic of China(NHFPC)launched the “National Preconception Health Care Project” (NPHCP) in 2010, focusing on rural areas and providing free PCC for the couples of reproductive age [19]. In this project, relevant preconception risk factors were classified according to their amenability to prevention and treatment. The objective of our study was to evaluate the impact of strategy and risk classification model of China’s NPHCP in identifying risk factors and stratifying the preconception health status of men and women of reproductive age.

## Methods

### Data source and study design

We conducted a secondary analysis of data collected within the framework of NPHCP during April 2010 to December 2012 to investigate the characteristics of preconception risk factors among married Chinese women and men of reproductive age. Methodological details of the project have been described previously [20–22]. Briefly, the study covered 220 counties in China. Selected rural counties in all provinces and the urban counties that wanted to participate in this project were included in this population-based prospective cohort study.

NHFPC established the implementation and quality control standards for this program [20, 21]. Local community staff investigated the conception plans of the couples, and those planning to conceive within the next six months were enrolled and invited to attend a free health examination. Professional doctors specially trained in obstetrics, genetic and other related specialties provided necessary medical advice to the couples. NHFPC has drafted and published the consultation guide for common preconception health problems. All couples enrolled accepted a free preconception health examination, risk evaluation, health education and medical advice based on the risk factors. A written informed consent was obtained from each participant, and this study was approved by the Institutional Review Board of the Chinese Association of Maternal and Child Health Studies [20, 21].

Preconception examination included (1) a medical history: current medical illness and use of any medication, family history of hypertension, diabetes, congenital or genetic diseases in the first-degree relatives, life style, dietary habits and exposure to environmental and occupational hazards; (2) physical examination: height, weight, blood pressure, heart rate, palpation of thyroid gland, auscultation of the heart and lungs, abdominal palpation, examination of the limbs and the spine; (3) clinical laboratory tests: genital swabs for microbiological culture and sensitivity, gonococcus and chlamydia test, hemoglobin and full blood count, urine for bacteriology and culture, blood type, serum glucose, liver, renal function and thyroid function tests, hepatitis B serology, syphilis test, TORCH (toxopasma, rubella virus, cytomegalovirus, and herpes simplex virus) screen, and gynecological ultrasound; (4) past medical history: hypertension, diabetes, cardiac diseases, immune system diseases, renal diseases and other chronic diseases; (5) past obstetric history including history of induced abortion, spontaneous abortion, live birth, stillbirth, neonatal death, fetal abnormality, preterm birth and multiple pregnancy. Trained staff regularly recorded and entered the information into the NHFPC database.

### Preconception risk evaluation and classification model

The aim of the preconception health examination was to identify all the risk factors as far as possible, and treat accordingly. Therefore, instead of assessing the degree of exposure, we developed a preconception risk classification system based on their amenability to prevention and treatment according to Preconception Health Examination and Risk Evaluation Guides (Science and Technology Division of NHFPC) (Table 1). Risk factors were categorized into five preconception risk classes: **A**-avoidable risk factors, **B**-benefiting from targeted medical intervention before conception, **C**-controllable but requiring close monitoring and treatment during pregnancy, **D**-diagnosable prenatally but the risk factor not modifiable preconceptionally, **X**-pregnancy not advisable. The couples with category X risk factor were advised to use appropriate contraception and were considered in further analysis. Participants with missing or incomplete records were excluded from analysis.

**Table 1:** Definition of “ABCDX” category of preconception risk factors

Category	Definition	Risk factors
A	Avoidable risks, i.e. they could be avoided though health education and eliminating work place hazards etc.	*Maternal:* smoking, alcohol consumption, exposure to toxins, radiation, noise, pesticide, organic solvent, heavy metal, inadequate nutrition (no intake of meat and egg, no intake of fresh vegetables, raw meat eating habit) *Paternal:* smoking, alcohol, consumption, exposure to toxins, radiation exposure, noise, pesticide, organic solvent, high temperature, preputial ring, inadequate nutrition (no intake of meat and egg, no intake of fresh vegetables, raw meat eating habit)
B	Benefiting from targeted medical intervention,	*Maternal:* anemia^a^, bacterial vaginitis, candida infection, gonorrhoea, trichomoniasis, Toxoplasma gondii infection (IgM positive), gingival hemorrhage, history of psychological disorder; *Paternal:* abnormal liver function, abnormal renal function, spermatic cord varicocele, hypertension, congenital heart disease, history of chronic renal disease history, cancer, epilepsy, or psychological disorder
C	Controllable risk factors, i.e. diseases and conditions that can’t be cured but risk can be modified and ameliorated. Close monitoring and medical supervision is required during the pregnancy	*Maternal:* Thrombocytopenia^b^, abnormal liver function, abnormal renal function, abnormal TSH, HBs-Ag positive, HBe-Ag positive, cytomegalovirus IgM positive, chlamydia positive, syphilis screening positive, Rh negative, history of gynecological diseases, preterm birth, diabetes, congenital heart disease, hypertension, malignancy, chronic renal disease, reported epilepsy, tuberculosis, use of narcotics; *Paternal*: HBs antigen positive, HBe antigen positive, syphilis screening positive, use of narcotics, thyroid disease
D	Diagnosable prenatally but risk factor is not modifiable preconceptionally i.e. women with these risk factors may benefit from preconception risk evaluation, counseling and prenatal diagnosis.	*Maternal:* Maternal birth defect, history of previous child with birth defects, mental retardation, history of recurrent abortion, stillbirth, or neonatal death, family history of Mediterranean anemia, G6PD deficiency, Albinism, Down’s syndrome, visual impairment; hearing impairment; *Paternal:* Paternal birth defect, mental retardation, family history of neonatal death, Mediterranean anemia, G6PD deficiency, Albinism, Down’s syndrome, hemophilia, family history of visual impairment or hearing impairment
X	Women with these risk factors are advised against pregnancy. Pregnancy should be evaluated under specialist after treatment.	Maternal: severe heart failure, severe thrombocytopenia^c^, severe anemia^d^

^a^Anemia referred to haemoglobin ranging from 60–109g/L

^b^Thrombocytopenia referred to platelet ranging from 50 to 100*10^9^/L

^c^Severe thrombocytopenia referred to platelet less than 50*10^9^/L

^d^Severe anemia referred to haemoglobin less than 60g/L

### Statistical analysis

Statistical analysis was performed using SPSS statistical software version 15.0 (SPSS, System for Windows, Chicago, USA). Data are presented as number (%) and mean ± standard deviation (SD). For comparing groups, we used independent samples *t*-test for continuous variable and *χ* ^2^ test for categorical variables. All *P*-values were two-tailed, and a *P* < 0.05 was considered to be statistically significant.

## Results

### General characteristics of the study population

During April 2010 to December 2012, a total of 22.42 million married Chinese couples planning to conceive within the six months were recruited to the study from 220 different counties. After excluding those with incomplete medical records and lost to follow-up, data from 2,142,849 couples were available for analysis. NPHCP targeted couples of reproductive age mainly from rural areas, and covered most areas, regions, and ethnicities from all provinces of mainland China. 92.36% couples were from rural areas and 89.2% women and 88.3% men had education below university level. Other socio-demographic details of the participants are presented in Tables 2 and 3.

**Table 2:** Socio-demographic characteristics of women in different preconception risk factor classification categories

		No risk factors	A	B	C	D	X
Age	≤25	24.7%	21.6%	26.3%	22.4%	14.7%	21.3%
	25–30	46.3%	47.4%	46.7%	44.1%	38.5%	46.0%
	30–35	19.5%	21.2%	18.2%	20.9%	27.1%^*^	20.1%
	≥35	9.6%	9.8%	8.7%	12.6%	19.8%^*^	12.6%^*^
Area	Rural area	93.9%	89.6%	94.0%	94.0%	94.3%	92.2%
Race	Han	92.7%	91.6%	92.5%	88.7%	84.0%^*^	84.2%^*^
Education	Secondary school or lower	71.5%	64.8%^*^	69.4%	71.8%	77.4%	75.5%
	High school	18.7%	19.7%	18.7%	17.1%	13.8%^*^	16.6%
	College or higher	9.8%	15.5%^*^	11.8%	11.1%	8.8%	8.0%

^*^ *P* value <0.05 compared with those women having no risk factors

**Table 3:** Socio-demographic characteristics of men in different preconception risk categories

		No risk factors	A	B	C	D	X
Age	≤25	9.2%	10.2%	10.3%	9.1%	4.5%^*^	-
	25–30	43.1%	44.5%	44.9%	45.5%	38.6%^*^	-
	30–35	28.8%	27.6%	28.0%	28.1%	33.0%^*^	-
	≥35	18.9%	17.6%	16.8%	17.3%	23.9%^*^	-
Area	Rural area	92.6%	92.1%	90.1%	92.2%	85.7%	-
Race	Han	92.7%	91.3%	88.4%	92.3%	91.1%	-
Education	Secondary school or lower	69.3%	69.0%	63.1%	66.4%	61.9%	-
	High school	20.0%	19.2%	20.1%	20.2%	16.9%	-
	College or higher	10.6%	11.8%	16.8%^*^	13.4%	21.3%^*^	-

^*^:*P* value <0.05 compared with those men having no risk factors

### Preconception risk factor classification

As demonstrated in Tables 2 and 3, category D risk was more common among couples in the age group 30–35 years and >35 years (*P* < 0.05). There were no significant differences between rural areas and cities in both couples in terms of risk factor categories. Proportionally, more women of non-Han ethnicity were classified in category D and X compared to those with no risk factors, while there was no difference in that ratio among men. Women with category A, and men with category B and D risk factors had higher education levels (*P* < 0.05).

### Distribution preconception risk factors

Distribution of the participants in different preconception risk categories is presented in Table 4. Among 2,142,849 couples, 46.25% women had preconception risks, mainly of category A, B and C. 9.80% women had category A risks including alcohol consumption (3.4%), inadequate protein intake (1.36%) and exposure to noise (1.18%). 14.83% women were had category B risks, such as anemia (8.40%), gingival hemorrhage (3.57%) and vaginitis (2.29%). Moreover, 23.5% of women had category C risks, such as thyroid dysfunction (6.34%), HBV infection (4.76%), history of gynecological diseases (3.41%) and/or category D risks, such as history of spontaneous abortion (2.66%) and adverse pregnancy history (1.12%). On the other hand, 51.92% of couples had paternal risks, and 38.13% of them had category A risk factors including alcohol (29.61%) and smoking (29.07%) (Table 4).

**Table 4:** Distribution of preconception risk factors among women and men in different preconception risk categories

Risk factors	Total number (%)	Risk factors	Total number (%)
Maternal		Paternal	
A			
Alcohol consumption	72,808(3.40%)	Alcohol consumption	634,547(29.61%)
No intake meat and egg	29,126(1.36%)	Smoking	622,834(29.07%)
Noise exposure	25,267(1.18%)	Preputial ring	84,659(3.95%)
Others^a^	82,739(3.86%)	Noise exposure	48,399(2.26%)
		Pesticide exposure	26,753(1.25%)
		No intake meat and egg	25,857(1.21%)
		Organic solvent exposure	24,211(1.13%)
		High temperature exposure	23,388(1.09%)
		Others^i^	41,492(1.94%)
B			
Anemia^b^	179,941(8.40%)	Abnormal liver function	152,862(7.13%)
Gingival hemorrhage	76,595(3.57%)	Abnormal renal function	26,107(1.22%)
Vaginitis^c^	49,162(2.29%)	Others^j^	9,973(0.47%)
Others^d^	12,257(0.57%)		
C			
Abnormal TSH	135,958(6.34%)	HBs antigen positive	135,958(5.87%)
HBs-Ag positive	101,970(4.76%)	HBe antigen positive	101,970(1.68%)
Gynecological diseases history	73,107(3.41%)	Others^k^	10,111(0.47%)
Abnormal renal function	57,680(2.69%)		
Abnormal liver function	54,192(2.53%)		
Thrombocytopenia^b^	31,522(1.47%)		
HBe-Ag positive	31,459(1.47%)		
Others^e^	132,582(6.19%)		
D			
Spontaneous abortion history	57,060(2.66%)	Others^l^	4,305(0.20%)
Adverse pregnancy history^f^	23,878(1.12%)		
Others^g^	8,873(0.41%)		
X			
Others^h^	5,259(0.25%)		

^a^including 20,647 not eating fresh vegetables (0.96%), 16,435 pesticide exposure (0.77%), 16,049 organic solvent exposure (0.75%), 12,540 radiation exposure (0.59%), 9,717 smoking (0.45%), 5,582 raw meat eating habit (0.26%) and 1,765 heavy metal exposure (0.08%)

^b^Anemia referred to hemoglobin ranging from 60–109g/L

^c^Vaginitis included 27,657 Candida infection, 11,398 Bacterial vaginitis and 10,107 Trichomonasis

^d^including 10,107 Trichomoniasis (0.47%), 7,672 Toxoplasma gondiiIgM positive (0.36%), 4,545Gonococcal infection (0.21%) and 40 history of psychological disease (0.00%)

^e^including 20,705 Rh negative (0.97%), 9,290 Cytomegalovirus IgM positive (0.43%), 9,266 Chlamydia positive (0.43%), 8,482 Syphilis screening positive (0.40%), 4,395 history of preterm birth (0.21%), 14,383 diabetes (0.67%), 1,830 reported hypertension (0.09%), 1,655 reported history of tuberculosis (0.08%), 1,392 anesthetic drug use (0.07%), 1,327 congenital heart disease (0.06%), 1,018 reported tumor history (0.05%), 897 reported chronic renal disease history (0.04%) and 882 reported epilepsy history (0.04%)

^f^Adverse pregnancy history included 16,824 with history of stillbirth and 7,054 with history of birth defects

^g^including 4,515 with birth defects (0.21%), 1,527 family history of neonatal death (0.07%), 1,416 mental retardation (0.07%), 923 family history of Mediterranean anemia (0.04%), 254 family history of G6PD deficiency (0.01%), 138 family history of Albinism (0.01%), 92 family history of Down’s syndrome (0.00%), 5 family history of hearing impairment (0.00%), 2 family history of mental retardation (0.00%) and 1family history of visual impairment (0.00%)

^h^including 2,707 severe thrombocytopenia (0.13%) and 2,552 severe anemia (0.12%). Severe thrombocytopenia referred to platelet less than 50*10^9^/L. Severe anemia referred to hemoglobin less than 60g/L

^i^including 18,726 not eating fresh vegetables (0.87%), 9,734 radiation exposure (0.45%), 9,454 raw meat eating habit (0.44%) and 3,578 exposure to heavy metals (0.17%)

^j^including 5,325 spermatic cord varicocele (0.25%), 2,432 hypertension (0.11%), 1,052 congenital heart disease (0.05%), 598 chronic renal disease history (0.03%), 404 epilepsy (0.02%), 159 history of cancer (0.01%) and 3 history of psychological disease (0.00%)

^k^including 7,771 Syphilis screening positive (0.36%), 1,348 anesthetic drug use (0.06%) and 992 reported thyroid disease (0.05%)

^l^including 2,344 with birth defects (0.11%), 658 family history of neonatal death (0.03%), 603 family history of Mediterranean anemia (0.03%), 274 family history of G6PD deficiency (0.01%), 248 mental retardation (0.01%), 138 family history of Albinism (0.01%), 78 family history of Down’s syndrome (0.00%), 4 family history of hearing impairment (0.00%), 1family history of hemophilia (0.00%) and 1family history of visual impairment (0.00%)

## Discussion

This nation-wide free preconception care project targeting rural areas in China used an integrated model of PCC including both women and men. A novel classification system was used to classify risk factors based on their amenability to prevention and treatment, which stratified couples in five different risk categories. More than 68% of couples with conception plans within the next six months had one or more risk factors, and nearly 40% of these risk factors could be potentially modified by intervention before or during pregnancy. Approximately 23% of risk factors among women were in category A and B, whereas among men the figure was 45%. Avoidable risk factors were more common among men compared with women suggesting that men may have riskier behavior than women, with almost 30% of men reporting consumption of alcohol and smoking.

Our study revealed that preconception risk evaluation in couples with plans to conceive within six months could be meaningful as nearly two-thirds of the recruited couples had preconception risk factors, and 23% maternal risk factors were in category A and B, and thereby potentially avoidable or modifiable preconceptionally by health education, medical intervention and life style changes. More importantly, a similar situation was observed regarding paternal risk factors. Almost 45% of the male partners consumed alcohol or smoked, which may lead to passive smoking by women, a fact often ignored in preconception care. Some European countries have preconception care recommendations for women with chronic diseases, such as diabetes and epilepsy, but guidelines are heterogeneous and recommendations for healthy women and men are fragmented and inconsistent [22]. Our results further enforce the need for an integrated approach to PCC that includes both women and men.

A more innovative and integrated approach to PCC for both women and men is needed for achieving optimal reproductive health status before pregnancy and better pregnancy outcomes [23, 24]. Preconception health promotion may be useful in eliminating some of the Category A and B risk factors before pregnancy. However, some risk factors, such as smoking, alcohol and substance abuse, would require longer term strategies to achieve sustained amelioration. A more comprehensive health promotion strategy during pregnancy would be required for managing other risk categories to achieve better pregnancy outcomes.

The preconception risk classification system used in this big population-based study was practical for stratifying preconception health status of the couples, and helpful in organizing targeted educational and health care interventions, and identifying need for referral. The risk classification was based on existing risk factors during the preconception period and categorized by whether it could be prenatally avoided or modified during the preconception period or prenatally. As preconception risks may vary from prenatal risks, Considering different methods and timing of intervention is important. Nearly half of the risk factors identified were avoidable or preventable by medical intervention during the preconception period in this study, allowing for a window of opportunity for personalized lifestyle modification and health care to achieve better pregnancy outcome. Despite the evidence supporting the value and importance of PCC [25], it is reported that there is lack of sufficient research attention to clinical PCC service delivery, and a more detailed consideration of the practicalities of implementing PCC within contemporary women’s health care is required [26]. This integrated universal free PCC service provided in rural China could be a promising model if its positive impact on pregnancy outcomes could be demonstrated in future.

Our study does have some limitations. Follow-up of risk modifications was not included in this study, so the impact of preconception risk classification on the health status of the couple could not be assessed. Prevalence of adverse pregnancy history and chronic disease history in couples planning pregnancy might have been underestimated as this was based on self-reporting and recall bias cannot be excluded.

## Conclusions

This project provided new insights into preconception health of Chinese couples of reproductive age. More than half of the male partners planning to father a child were exposed to risk factors during the preconception period, suggesting that an integrated approach to PCC including both women and men is justified. Stratification based on the new risk classification model demonstrated that a majority of the risk factors are avoidable or preventable by medical intervention. Therefore, universal free PCC can be expected to improve pregnancy outcomes in rural China.

## Abbreviations

LBW: Low birth weight
NHFPC: The National Health and Family Planning Commission of the People’s Republic of China
NPHCP: National Preconception Health Care Project
PCC: Preconception care
